# Characterization of Reproductive Microbiota of Primiparous Cows During Early Postpartum Periods in the Presence and Absence of Endometritis

**DOI:** 10.3389/fvets.2021.736996

**Published:** 2021-10-18

**Authors:** Hayami Kudo, Tomochika Sugiura, Seiya Higashi, Kentaro Oka, Motomichi Takahashi, Shigeru Kamiya, Yutaka Tamura, Masaru Usui

**Affiliations:** ^1^Department of Health and Environmental Sciences, School of Veterinary Medicine, Rakuno Gakuen University, Hokkaido, Japan; ^2^Research Department, R&D Division, Miyarisan Pharmaceutical Co., Ltd., Saitama, Japan; ^3^Department of Large Animal Clinical Science, School of Veterinary Medicine, Rakuno Gakuen University, Hokkaido, Japan

**Keywords:** endometritis, reproductive tract microbiota, *Trueperella pyogenes*, early postpartum periods, probiotics

## Abstract

Endometritis has a major impact on fertility in postpartum dairy cows. Since previous studies showed an association between reproductive microbiota and perinatal disease, we monitored both bovine uterine and vaginal microbiota in primiparous cows to elucidate the effect of early postpartum microbiota on endometritis. Uterine and vaginal samples were collected at time points from pre-calving to 35 days postpartum (DPP), and analyzed by 16S rRNA sequencing, combined with ancillary bacterial culture. A total of seven healthy cows and seven cows diagnosed with endometritis on 35 DPP were used in the current study. The uterine and vaginal microbiota showed a maximum of 20.1% shared amplicon sequence variants (ASVs) at linked time points. 16S rRNA based analysis and traditional culture methods revealed that *Trueperella* showed a higher abundance in both uterus and vagina of the endometritis group compared to the healthy group on 21 DPP (*U*-test *p* < 0.05). Differential abundance analysis of the uterine microbiota showed that *Enterococcus* and six bacterial genera including *Bifidobacterium* were unique to the healthy group on the day of calving (0 DPP) and 28 DPP, respectively. In contrast, *Histophilus* and *Mogibacteriaceae* were characteristic bacteria in the vagina pre-calving in cows that later developed endometritis, suggesting that these bacteria could be valuable to predict clinical outcomes. Comparing the abundances of bacterial genera in the uterine microbiota, a negative correlation was observed between *Trueperella* and several bacteria including *Lactobacillus*. These results suggest that building an environment where there is an increase in bacteria that are generally recognized as beneficial, such as *Lactobacillus*, may be one possible solution to reduce the abundance of *Trueperella* and control endometritis.

## Introduction

Endometrial inflammation is common in postpartum dairy cows (1). Persistent uterine inflammation is clinically defined as endometritis. This modulates ovarian function and has a negative impact on fertility, resulting in high economic losses (2). The gold standard for its diagnosis after 21 days postpartum (DPP), is palpation and scoring by vaginal mucus and polymorphonuclear leukocyte (PMN) count (3, 4). Antimicrobial agents including disinfectants are routinely used in the treatment of endometritis. However, emergence and spread of antimicrobial resistance (AMR) has become a global concern for both human and livestock animal health, and appropriate use of antimicrobial agents has been strongly advocated in the veterinary field following WHO 2015 Global Action Plan on AMR, and new methods of prevention or treatment of endometritis are required (5, 6).

Recently, it has been shown that the uterus is not sterile during pregnancy and has its own microbiome (7), and non-commensal bacteria from the environment are thought to rapidly colonize the uterus shortly after calving (8). Since bacteria can be isolated from the uterus of approximately 80% of cows within 21 DPP, previous studies have focused on the late postpartum period, the time after which endometritis is established (9). After 21 DPP, *Escherichia coli, Trueperella pyogenes*, and *Fusobacterium necrophorum* are frequently isolated from cows with endometritis (4, 10, 11). These bacteria may interact with each other and contribute to establish the complex pathological processes of endometritis.

The uterine microbiota of the bovine postpartum period has previously been explored by culture-dependent methods. However, it is difficult to isolate bacteria with slow growth or low bacterial counts, and optimization of culture conditions is needed (12–14). With the recent progression of sequencing technology, temporal changes of uterine and vaginal microbiota in the postpartum period have been analyzed (15–18). These studies suggested that microbiota do impact on reproductive disease, although the studies did not differentiate primiparous and multiparous cows. The uterine and vaginal microbiota of multiparous cows are thought to be affected by previous parturitions (19) and therefore, studies for primiparous cows that have never experienced calving and subsequent bacterial contamination are required. In this study, to further clarify the relationship between early postpartum genital tract microbiota and endometritis, the uterine and vaginal microbiota of primiparous cows with or without endometritis were compared by sampling over the time period from pre-calving to 35 DPP.

## Materials and Methods

### Animals and Diagnosis of Endometritis

This study was conducted according to the institutional guidelines for animal experiments of Rakuno Gakuen University (approval no. VH16C7). Seventeen primiparous cows (Holstein Friesian, range of age at first calving 22–27 months), received artificial insemination (AI) or embryo transfer (ET), were enrolled from December 2017 to August 2018 at the Rakuno Gakuen University for this study. All cows were diagnosed as either healthy or suffering from endometritis at 35 DPP, according to the methods previously reported (4). Briefly, clinical endometritis was diagnosed with vaginal discharge scoring as follows: 0 = clear or translucent mucus; 1 = mucus containing flecks of white or off-white pus; 2 = exudate containing <50% white or off-white mucopurulent material; and 3 = exudate containing ≥50% purulent material, usually white or yellow, occasionally bloody. The degree of recovery of the uterus was also assessed by rectal examination as follows: 0 = the diameter of largest uterine horn ≤3.5 cm and cervical diameter ≤4.5 cm; 1 = the diameter of largest uterine horn more than 3.5 to <5.5 cm and cervical diameter more than 4.5 to <7.0 cm; 2 = the diameter of largest uterine horn ≥5.5 cm and cervical diameter ≥7.0 cm. The numbers of epithelial endometrial cells and PMNs were counted to assess subclinical endometritis (3). Endometritis was defined either as total gynecological examination scoring >1, or a PMN ratio >10%. Three cows were excluded due to the following reasons: treatment with antibiotics, abnormal deliveries, and urovagina. The criteria for endometritis, scores and sampling dates of the 14 cows (seven healthy cows and seven cows diagnosed with endometritis at 35 DPP) are summarized in Supplementary Figure 1 and Supplementary Table 1. In addition, parturitions were scored for difficulty and obstetric assistance required as follows: 1 = no assistance (*n* = 6); 2 = slight assistance with only one person (*n* = 5); 3 = moderate assistance with 2–3 persons (*n* = 1); 4 = severe dystocia with veterinary treatment (*n* = 2); 5 = cesarean section (*n* = 0).

### Sample Collection

Both uterine and vaginal samples were collected at the same time as follows; Pre (pre-calving, only for vaginal samples), 0 DPP (within 12 h after calving and after expulsion of the placenta), 7, 21, 28, and 35 DPP (Supplementary Table 1).

The perineum and vulva were cleaned with a 70% ethanol swab and wiped with a paper towel. Uterine samples were collected using the cytobrush technique (3). The cytobrush device (Metribrush; Fujihira Kogyo Inc., Tokyo, Japan), double-guarded with sterilized plastic sleeve and tube, was inserted through the vagina and reached the cervix guided by palpation per rectum. Then the plastic sleeve was pulled back and the brush was moved forward and rolled over the endometrium of the uterus body. The cytobrush was retracted into the tube in uterus, then retrieved through the vagina. The tip of the cytobrush was then cut using sterile scissors, placed in 5 ml sterile saline solution and vortexed vigorously. Vaginal samples were obtained by washing methods as previously described (20), as sampling by brushing of the vaginal fornix was not suitable to extract sufficient bacterial DNA. In brief, 50 ml of sterile saline was injected into the vagina, then flushed back 2 or 3 times. Within 1 h, vaginal washings were centrifuged at 8,000 × g for 15 min and pellets were re-suspended in 5 ml sterile saline. After vortexing, 500 μl of uterine and vaginal samples were used for the culture-based method and the remaining samples were mixed in an equal volume of sterile saline containing 40% glycerol. Mixtures were flash frozen in liquid nitrogen, and stored at −80°C until use.

### DNA Extraction

Bacterial DNA was extracted as described previously (21). After thawing, 2 ml of uterine and vaginal samples were mixed with 20 ml of sterile saline solution and centrifuged at 8,000 g for 15 min. Pellets were then suspended in 800 μl 10 mM Tris-HCl and 10 mM EDTA buffer containing lysozyme (Sigma-Aldrich Co., LCC, Missouri, USA) (Final concentration: 15 mg/ml). Mixtures were incubated at 37°C for 1 h. Purified achromopeptidase (Wako Pure Chemical Inc., Osaka, Japan) (Final concentration: 2,000 U/ml) was added and incubated 37°C for 30 min. Finally, proteinase K (Takara-Bio Inc., Shiga, Japan) (Final concentration: 1 mg/ml) with 20% sodium dodecyl sulfate (Sigma-Aldrich Co., LCC) was added and incubated at 55°C for 1 h. The DNA was isolated with phenol:chloroform:isoamyl alcohol (25:24:1, v/v), washed twice with 75% ethanol, and dissolved in 100 μl TE buffer. RNase A (Nippon Gene Co., Ltd., Tokyo, Japan) (Final concentration: 0.1 mg/ml) was added to the mixture, and incubated at 37°C for 1 h. Subsequently, the DNA was purified using a High Pure PCR Template Kit (Roche Inc., Basel, Switzerland) according to the manufacturer's instructions. Elution was performed in 50 μl of TE buffer and then samples were stored at −20°C until further analysis.

### 16S rRNA Gene Amplification and Sequencing

Extracted DNA was amplified, targeting the V3–V4 region of the bacterial 16S rRNA gene. The specific universal primer pair 341F (5′-CCTACGGGNGGCWGCAG) and 805R (5′-GACTACHVGGGTATCTAATCC) was used in this study (22). This primer set included the Illumina MiSeq sequencing adapter (forward primer: AATGATACGGCGACCACCGAGATCTACAC; reverse primer: CAAGCAGAAGACGGCATACGAGAT) and a unique barcode sequence that allowed the samples to be pooled for Illumina MiSeq sequencing. Polymerase chain reaction (PCR) was performed using MightyAmp DNA Polymerase (Takara-Bio Inc.) for 35 cycles. The PCR products were quality-checked on a 2% agarose gel electrophoresis and subsequently purified using SPRIselect beads (Beckman-Coulter Inc., California, USA). The amplified DNA was quantified using a ONEdsDNA System (Promega, Madison, WI, USA) and Quantus fluorometer (Promega). The PCR amplicon libraries were prepared by pooling approximately equal amounts of amplified DNA and sequenced on an Illumina MiSeq platform (Illumina, San Diego, USA), using the 2 × 300, v3 600-cycle kit (Illumina).

### Data Processing and Analysis

Illumina Miseq fastq raw reads were analyzed with QIIME 2 platform version: 2020.2 with default scripts (23). Sequences were demultiplexed and processed by the DADA2 program (24). Before merging, reads were trimmed according to quality threshold and adapter length (Forward: 280 bp, Reverse: 220 bp). After quality filtering steps (DADA2), total, average, minimum and maximum number of non-chimeric reads were 1,901,611, 14,086, 7,565, and 17,893 reads, respectively. Amplicon sequence variants (ASVs) obtained after DADA2 [more accurate than traditional operational taxonomic units (OTUs)] were assigned using the *qiime* feature-classifier classify-sklearn method (25) against the Greengenes database (version 13.8) with 99% similarity. For diversity analysis, the number of sequence reads was rarefied to the minimum sample reads (7,565 reads) using the *qiime* diversity core-metrics-phylogenetic method (23). The rarefaction curve confirmed that the sub-sampling was sufficient to detect the bacteria species in all samples. The alpha-diversities were calculated using the Simpson and Shannon index. Beta-diversity analysis, represented by principal coordinate analysis (PCoA), was applied to the resulting weighted distance matrices to generate two-dimensional plots. For differential abundance analysis between healthy and endometritis groups, random subsampling of sequence reads was not performed.

Statistical differences in alpha-diversities between healthy and endometritis groups at sampling time points were tested using the Mann-Whitney *U*-test. The significance of the groups in the community structure was tested using permutational multivariate analysis of variance (PERMANOVA). Relative abundances of genera between healthy and endometritis groups was tested using Mann-Whitney *U*-test. To identify unique microbial genera for both healthy and endometritis groups, ANOVA-Like Differential Expression 2 (ALDEx2), estimates of the composition of biological features from the number of reads transformed to central log ratio (clr), was performed (26, 27). The correlations between the abundances of genera in uterine and vaginal microbiota were determined using Spearman's rank correlation coefficient. A linear discriminant analysis (LDA) effect size (LEfSe) (28) approach was used to identify bacterial taxonomy that was significantly different between the “before birth” group (Pre) and “after birth” group (0 DPP) for both healthy and endometritic cows.

### Culture-Based Analysis

All samples used for DNA extraction were also cultured under aerobic and anaerobic conditions within 1 h from sampling. Tryptic soy (TS) agar (BD Bacto, New Jersey, USA) and blood liver (BL) agar (Nissui, Tokyo, Japan), which are widely used culture media in the analysis of human fecal microbiota (29) were adopted in this study. Both TS agar and BL agar were supplemented with 5% defibrinated horse blood. Briefly, for aerobic culture, 50 μl of 10-fold serial dilutions of sample suspensions were spread on TS agar and incubated at 37°C for 48 h. The same suspensions were spread on BL agar and incubated at 37°C for 48 h under anaerobic conditions (10% H_2_, 10% CO_2_, and 80% N_2_) using an anaerobic chamber. After incubation, the numbers of colonies with the same morphology on agar were counted. For several different types of colonies, a maximum of three colonies with the same morphology on one plate were picked from each agar and identified using MALDI-TOF MS (Bruker, Bremen, Germany). To arbitrate the results of MALDI-TOF MS and identify bacteria to species level, 16S rRNA gene sequencing was performed by using the universal primer pair 27F (5′-AGAGTTTGATCCTGGCTCAG) and 1492R (5′-GGTTACCTTGTTACGACTT) if bacterial strains obtained an identification scoring of <2.0 (30). The obtained sequences were compared using the basic local alignment search tool (BLAST) with the National Center for Biotechnology Information (NCBI) databases. A species result was adopted only when all three colonies had the same identification result. Mann-Whitney *U*-test was used to calculate significant differences in the number of bacteria, converted to log CFU/ml of samples, between healthy and endometritis groups.

## Results

### Alpha and Beta Diversities

**Figure 1:**
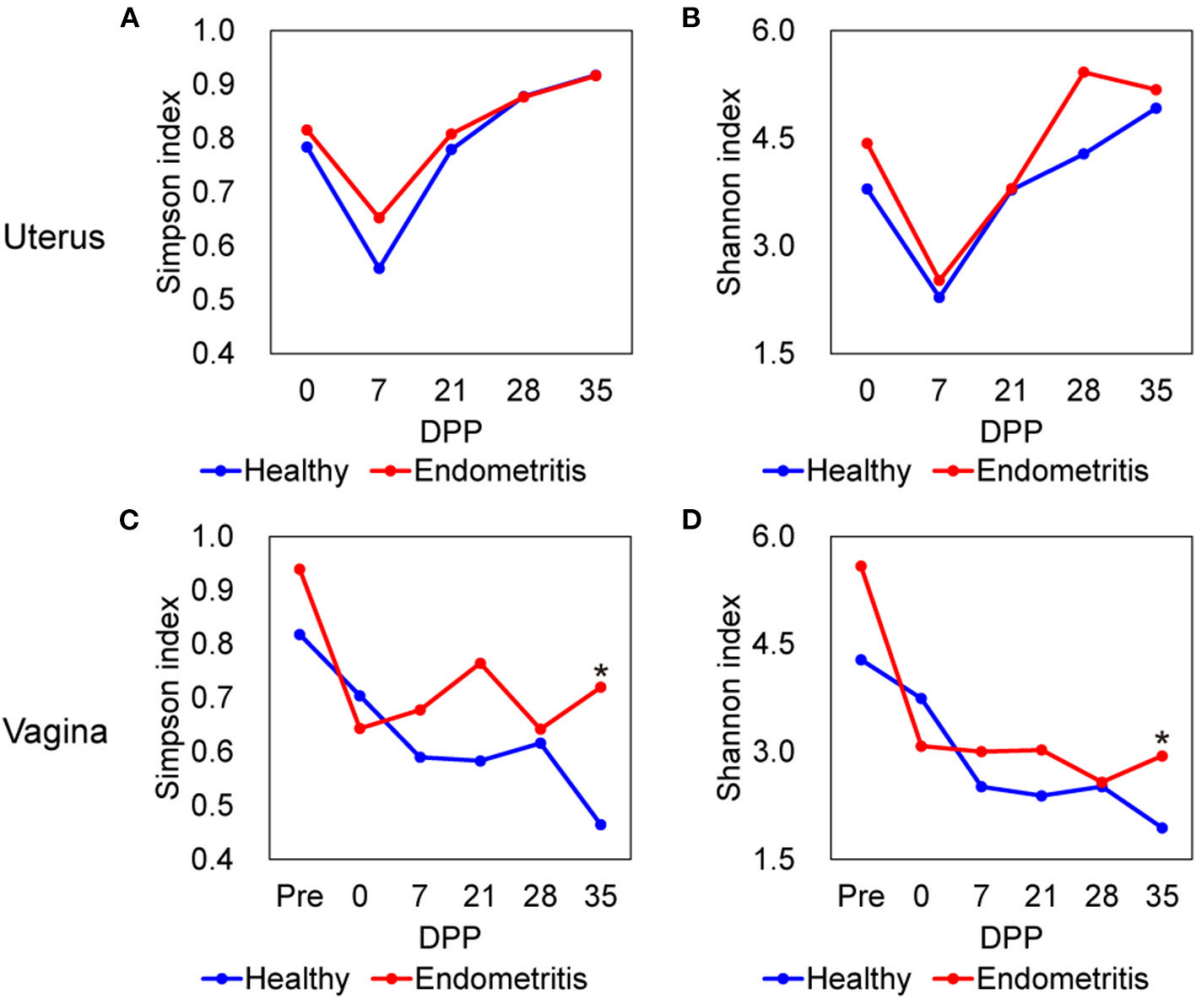
Alpha diversity of uterine **(A,B)** and vaginal **(C,D)** microbiota in healthy (blue) and endometritis (red) groups over 35 days postpartum (DPP). **(A,C)**, Simpson index; **(B,D)**, Shannon index; * indicates significant difference at *p* < 0.05 by Mann-Whitney *U*-test.

The alpha-diversities of both uterine and vaginal microbiota of the healthy and endometritis groups were measured using Simpson and Shannon indices (Figure 1). As shown in Figure 1, Simpson and Shannon indices of vaginal microbiota at 35 DPP were found to be significantly higher in the endometritis group than in the healthy group (Mann-Whitney *U*-test, *p* < 0.05). As a common trend within the healthy and endometritis groups, vaginal diversity was the highest before calving and tended to decrease as time passed (Mann-Whitney *U*-test, *p* < 0.01; Pre vs. 35 DPP), while uterine diversity was lowest at 7 DPP and recovered as time passed (Mann-Whitney *U*-test, *p* < 0.05; 7 vs. 0 DPP and 7 vs. 35 DPP).

Principal coordinate analysis based on weighted UniFrac distance showed that the uterine microbiota of the healthy group was clustered and separated from those of the endometritis group at 28 and 35 DPP (PERMANOVA, *p* < 0.05) (Supplementary Figure 2).

### Bacterial Communities in the Uterus and Vagina

**Figure 2:**
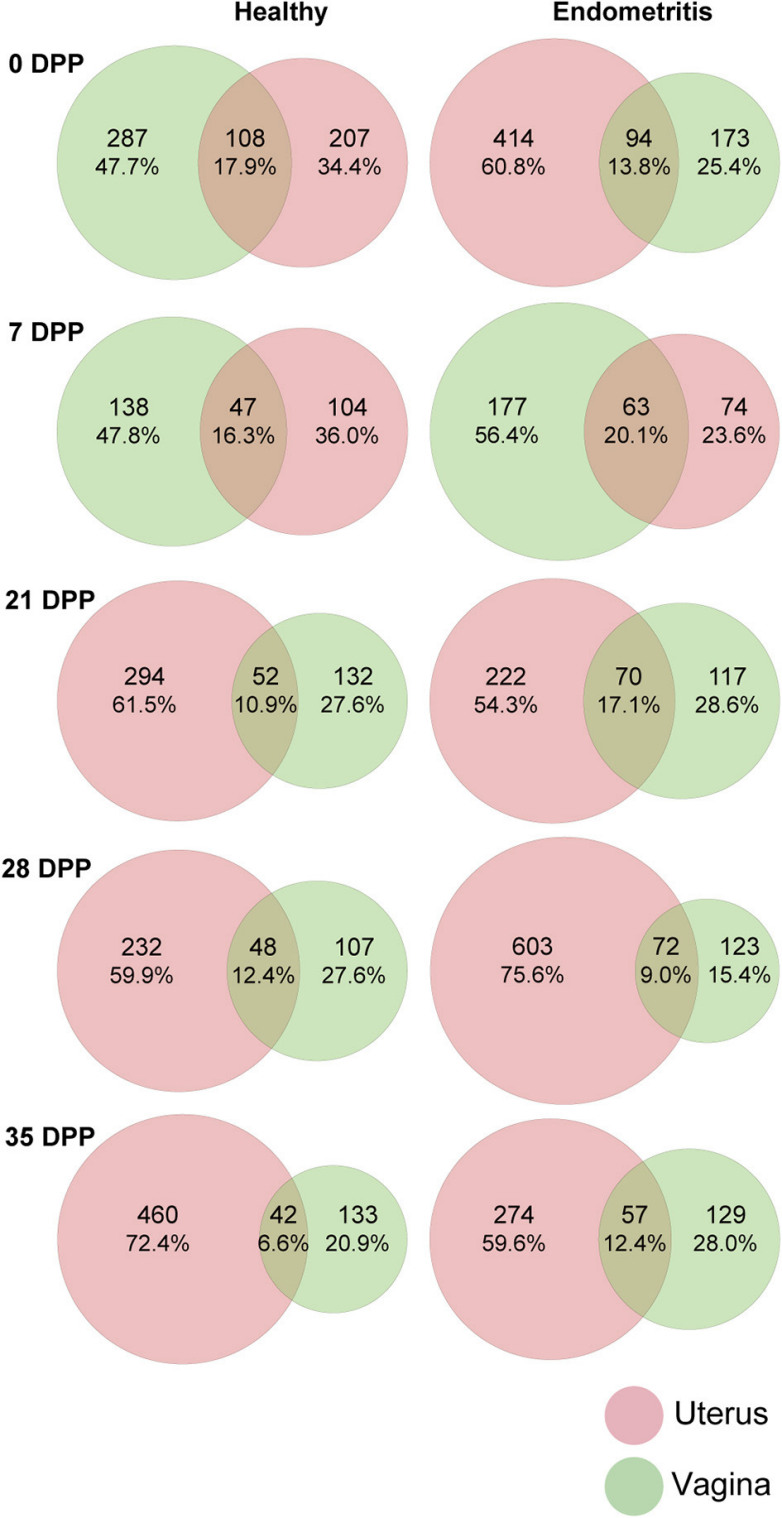
Venn diagram of amplicon sequence variants (ASVs) of uterine (red) and vaginal (green) microbiota over 35 days postpartum (DPP). Number of ASVs in each compartment and shared ASVs between uterine and vaginal microbiota are shown after removal of <0.1% of all observed leads.

A total of 3,918 ASVs were observed in samples. The ASVs shared between the uterine and vaginal microbiota of all animals at each time point are presented in Figure 2. Only 6.6–17.9 and 9.0–20.1% of all ASVs were shared between uterine and vaginal samples for the healthy group and endometritis group, respectively. In both the healthy and endometritis groups, the number of ASVs was higher in the vagina than in the uterus for 7 DPP. This trend was reversed after 21 DPP, namely the number of ASVs in the uterine samples was higher than that in the vaginal samples. In the healthy group, the number of ASVs at 0 DPP was almost the same in the uterine samples (207 ASVs, 34.4%) and vaginal samples (287 ASVs, 47.7%), whereas the number of ASVs in the uterine samples (414 ASVs, 60.8%) was higher than that in the vaginal samples (173 ASVs, 25.4%) in the endometritis group.

### Taxonomic Composition of Uterine and Vaginal Bacterial Communities

**Figure 3:**
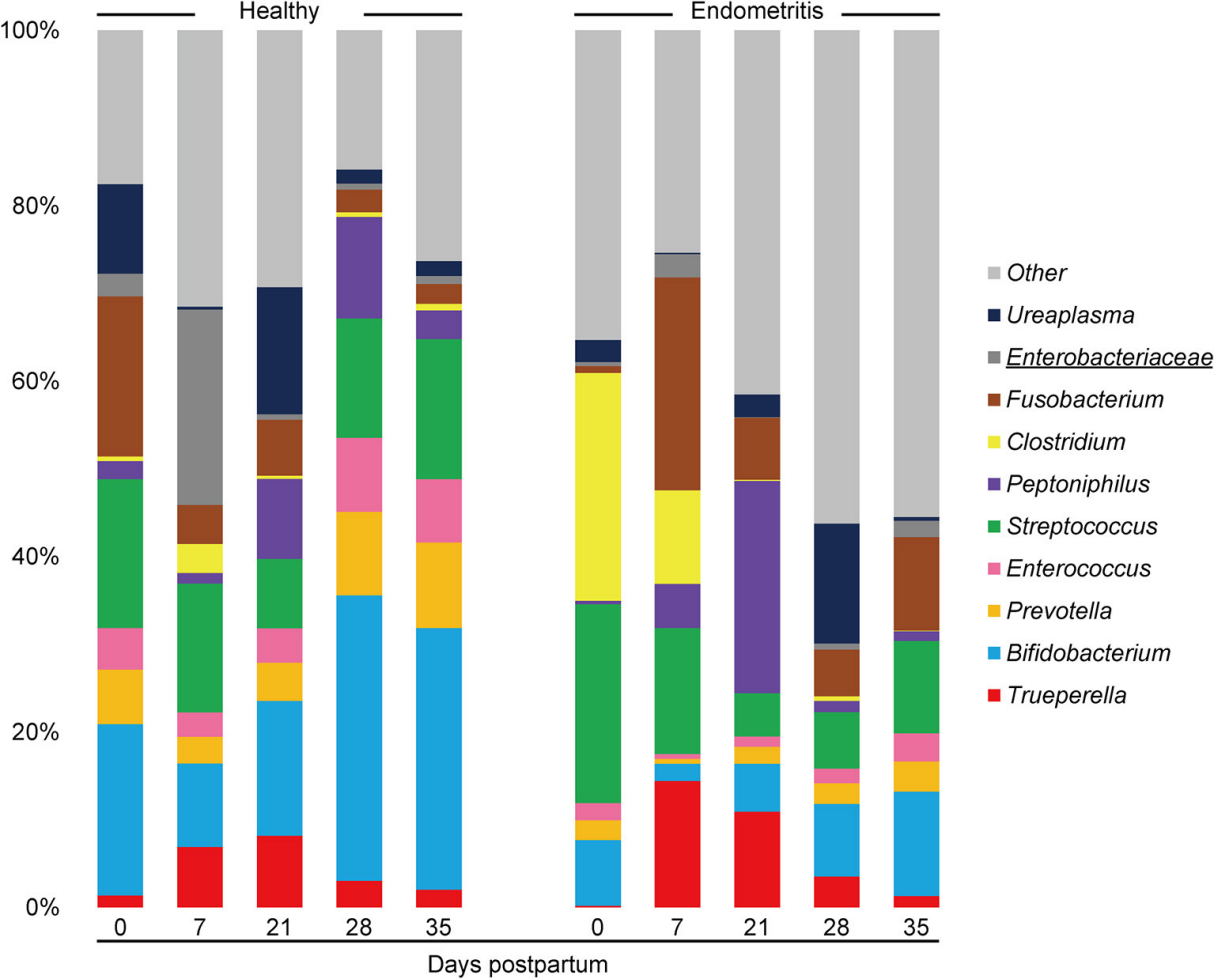
Stacked bar chart showing mean relative abundance of 10 most abundant genera in uterine microbiota of healthy and endometritis cows. The underlined legends indicate classification only at the family level with genus not precisely defined.

The mean relative abundances of the top 10 bacterial genera in the uterus is displayed by bar chart (Figure 3). The results showed that uterine bacterial communities in the healthy group during the study period were dominated by genus *Bifidobacterium* (The relative abundance of genus *Bifidobacterium* at 0, 7, 21, and 35 DPP were 19.5, 11.5, 20.0, 31.4, and 36.4%, respectively) followed by *Streptococcus* (The relative abundance of genus *Streptococcus* at 0, 7, 21, and 35 DPP were 17.0, 17.8, 10.3, 13.1, and 19.5%, respectively). In contrast, genus *Clostridium* was frequently detected and dominated at 0 DPP in the endometritis group (25.9%). The genus *Trueperella* was detected in both healthy and endometritis cows. With the exception that the genus *Ureaplasma* was most frequently predominant in the healthy group, vaginal bacterial communities exhibited the same dynamics as uterine in terms of genus *Bifidobacterium, Trueperella*, and *Clostridium* (Supplementary Figure 3).

These bacterial dynamics of the uterine and vaginal microbiota were also confirmed by traditional culture-dependent methods. A total of 1,213 colonies were isolated from 14 cows (healthy cows and cows with endometritis), representing 37 bacterial genera. For the three bacterial genera with the highest detection rates, bacteria count data were calculated from the number of colonies diluted 10-fold and converted to log CFU/ml of samples (Supplementary Table 2). As shown in Supplementary Table 2, the number of *T. pyogenes* was significantly higher in the endometritis group compared to the healthy group at 21 DPP in uterus (N.D. vs. 4.64 ± 0.23 log cfu/ml, *p* < 0.05) and vagina (2.62 ± 0.76 vs. 5.03 ± 0.27 log cfu/ml, *p* < 0.05), and at 35 DPP in the vagina (2.62 vs. 4.37 ± 0.73 log cfu/ml *p* < 0.05).

### Differential Abundance Analysis

To verify the difference in genera between the healthy and endometritis groups, differential abundance analysis was performed using ALDEx2, which estimates the composition of biological features in the sample based on the number of reads.

**Figure 4:**
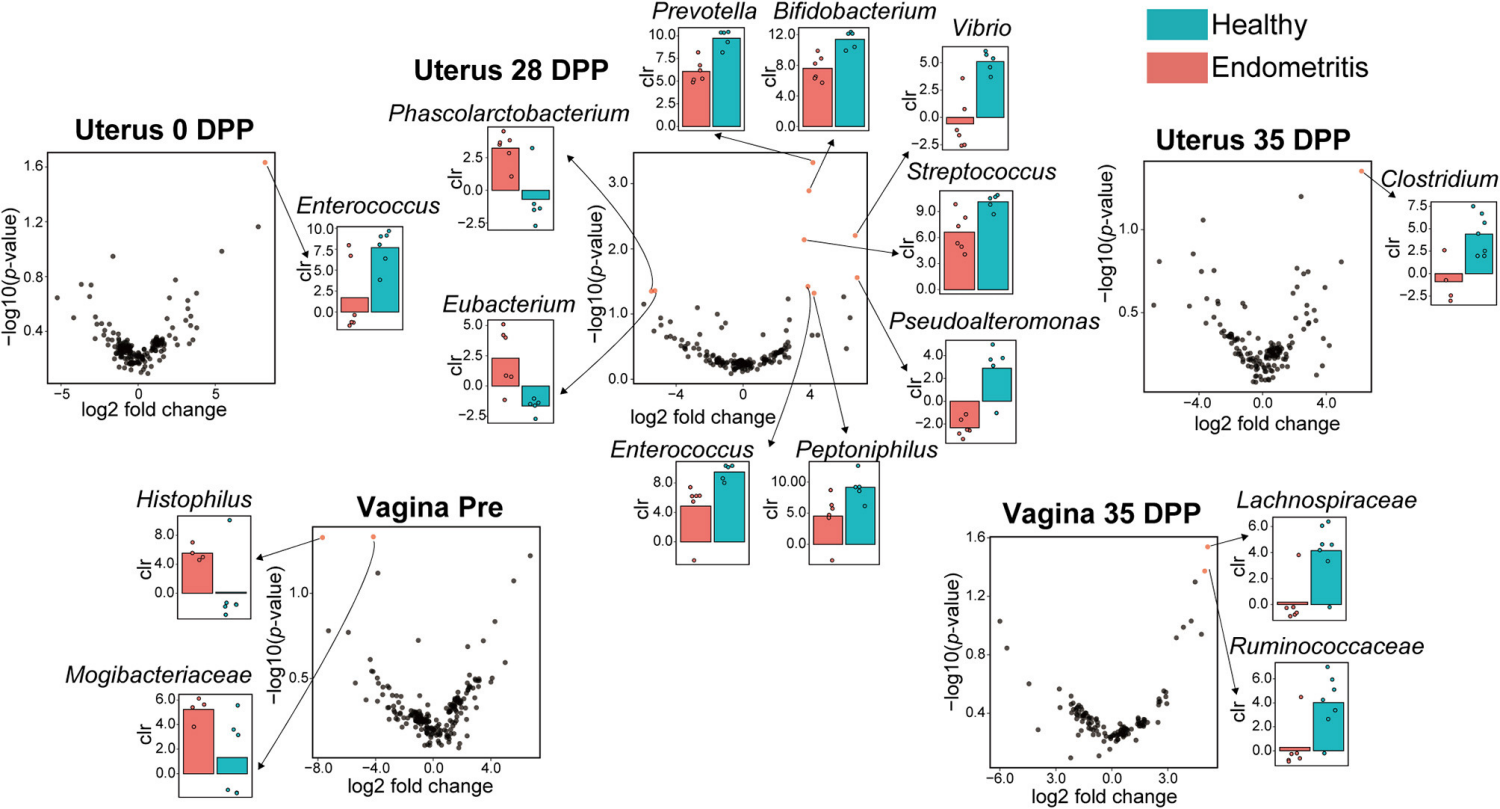
Volcano plot showing significantly different genera between the healthy and endometritis groups at each of the indicated days postpartum. Red plots represent significant hits (*p* < 0.05). The centered log ratio (clr) values of each bacterial genus for healthy (blue) and endometritis (red) groups, which correspond to the red plots, are shown on the bar chart.

Except for duplications, in total 10 of the bacterial genera were found to be different between the healthy and endometritis groups in uterine samples (Figure 4). The healthy group had higher abundances of genus *Enterococcus* at 0 DPP, and genus *Clostridium* at 35 DPP (*p* < 0.024 and *p* < 0.045, respectively). Genera differences were most detected at 28 DPP: *Prevotella, Bifidobacterium, Vibrio, Streptococcus, Pseudoalteromonas, Peptoniphilus*, and *Enterococcus* were significantly increased in the healthy group with *p* < 0.0005 to *p* < 0.049, whereas *Phascolarctobacterium* and *Eubacterium* showed a significant increase in the endometritis group (*p* < 0.044 and *p* < 0.045, respectively).

For vaginal samples, genus *Histophilus* and an unclassified genus of the family *Mogibacteriaceae* were found to be significantly increased in the endometritis group at pre-calving (*p* < 0.048 and *p* < 0.047, respectively), whereas unclassified genera of the families of *Lachnospiraceae* and *Ruminococcaseae* were significantly increased in the healthy group at 35 DPP (*p* < 0.029 and *p* < 0.043, respectively). There were no significant differences in the genera between healthy and endometritis groups at 0–28 DPP for vaginal samples, and 7–21 DPP for uterine samples.

### Correlation of Uterine Bacterial Communities

**Figure 5:**
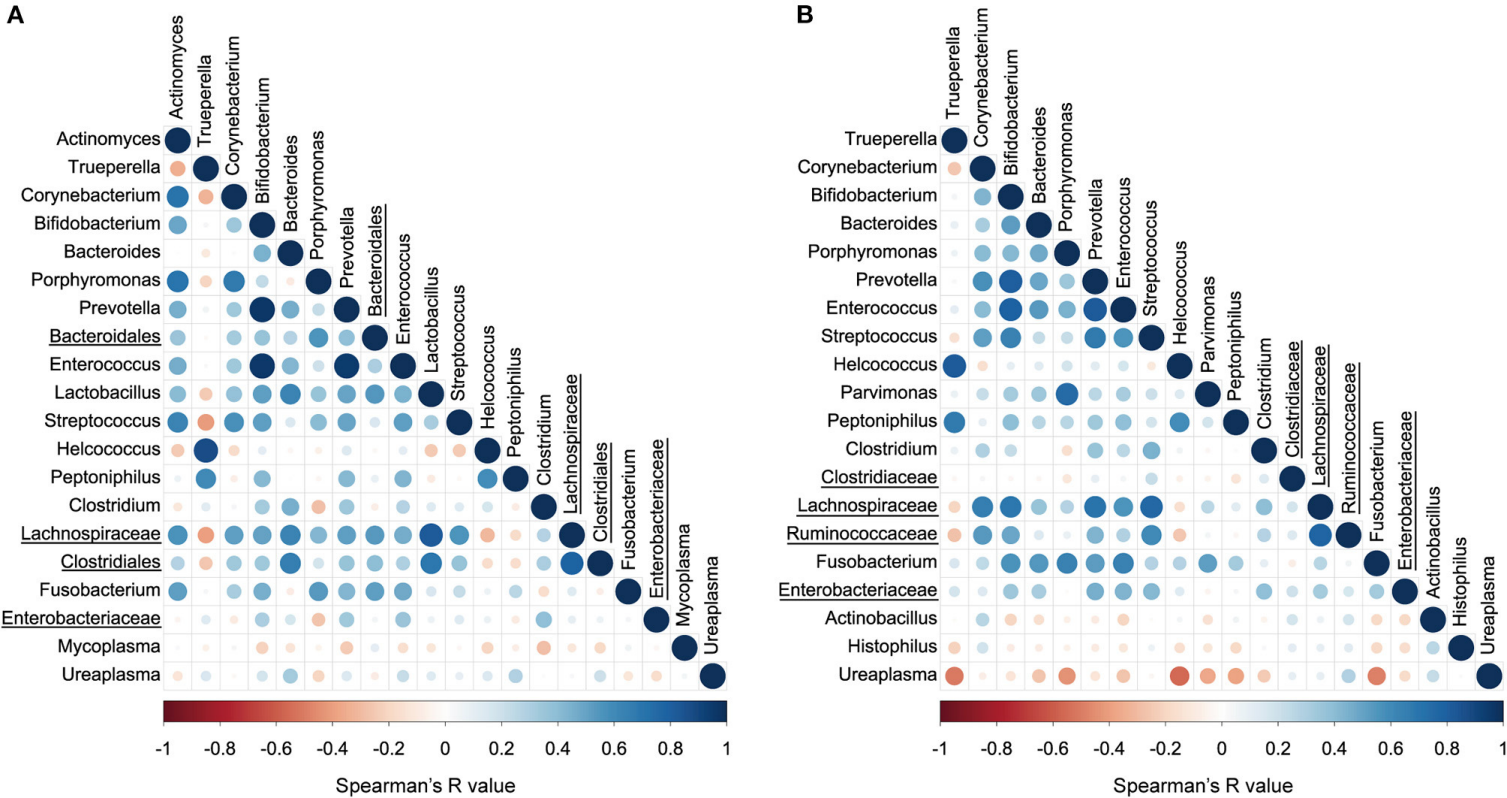
Correlation plot of top 20 abundant bacterial genera in uterine **(A)** and vaginal microbiota **(B)**. The colors and circle sizes depict the degree of Spearman's rank correlation coefficient (rho) and *p*-value, respectively. The underlined legends indicate classified only at the order level or family level with the genus not precisely defined.

The correlations between the top 20 abundant bacterial genera of the uterine and vaginal microbiota were analyzed mixing both healthy and endometritis groups (Figure 5A). There was a significantly positive correlation between *Trueperella* and *Helcococcus* (*r* = 0.875; *p* < 0.001) but significantly negative correlations with *Corynebacterium, Porphyromonas, Lactobacillus, Streptococcus*, unclassified genus of the family *Lachnospiraceae*, and unclassified genus of the order *Clostridiales*. The genus *Bifidobacterium* was shown to have strong positive correlations with the genera *Prevotella* and *Enterococcus* (*r* = 0.964 and 0.954, respectively; *p* < 0.001), and moderately positive correlations with *Lactobacillus* and *Streptococcus* (*r* = 0.519 and 0.513, respectively; *p* < 0.01). In the vaginal microbiota, there were statistically significant positive correlations between the genera *Trueperella* and *Helcococcus* (*r* = 0.816; *p* < 0.001), as well as between *Trueperella* and *Peptoniphilus* (*r* = 0.679; *p* < 0.001), whereas *Trueperella* showed moderately negative correlations with unclassified genus of the family *Lachnospiraceae* and *Ruminococcaseae* (*r* = −0.202 and −0.275, respectively; *p* < 0.01) (Figure 5B). The genus *Ureaplasma* was the most predominant and negatively correlated with many other bacteria.

### LEfSe Analysis on the Effect of Birth

For healthy cows, LEfSe analysis resulted in five genera which were significantly discriminative between “before birth” group (Pre) and “after birth” group (0 DPP) (>3 log 10 LDA score, *p* < 0.05) (Supplementary Figure 4). Genera *Helcococcus, Blautia*, and *Klebsiella* were represented in “before birth” group, while genus *Sutterella* and an unclassified genus of family *Alcaligenaceae*, were represented in “after birth” groups. By contrast, nine identified bacterial taxa including genera *Corynebacterium, Helcococcus*, an unclassified genus of the family *Veillonellaceae* were unique to “before birth” group of endometritic cows (>4 log 10 LDA score, *p* < 0.05).

## Discussion

Postpartum dairy cows are at high risk for a variety of diseases. There is some evidence that many risk factors are closely intertwined with endometritis, such as a negative energy balance, physiologic uterine inflammation, and the presence of bacteria in the environment and inside the genital tract (1, 31). In this study, we compared both uterine and vaginal microbiota of primiparous cows during early DPP using 16S rRNA gene-based metagenomic analysis in groups of cows with and without endometritis.

This study showed that the uterine microbiota at 7 DPP contained the lowest bacterial richness regardless of the presence or absence of endometritis. Uterine mucosa became rapidly colonized with environmental bacteria after delivery (8), and the number of PMNs also increased with the initial host immune response, peaking at 14 DPP (32). Therefore, these low microbial diversities at 7 DPP may be as a result of physiological inflammation which expel detritus. In order to understand the pathogenesis of endometritis it is important to clarify which pathogenic bacteria can consequently colonize the endometrium after 7 DPP. In contrast to the uterine microbiota, the vaginal microbiota had the highest richness before calving and decreased with time. Unlike humans, whose vaginal flora is usually dominated by genus *Lactobacillus*, those of healthy cows is composed of a variety of bacteria depending on the estrous cycle (33, 34). Therefore, it was expected that the bacterial composition of the vagina would not yet be fully restored to its prepartum state at 35 DPP. The diversity of the vaginal microbiota was higher in the endometritis group than in the healthy group at 35 DPP, however there was no difference in beta-diversities (PERMANOVA *p* = 0.22).

The uterine and vaginal microbiota did not share more than 20.1% of all ASVs within the coverage period (from 0 to 35 DPP). Previous reports have shown that the postpartum uterine and vaginal microbiota shared 9.2% core OTUs (20), suggesting that each of the two compartments may be composed of independent populations of bacteria. The number of ASVs in the uterine and vaginal microbiota of healthy cows on 0 DPP was 207 and 287, respectively, whereas those of endometritis group were 414 and 173, suggesting that the presence of a large number of bacteria in the uterus at 0 DPP may have an effect on subsequent prognosis.

At the genus level, *Bifidobacterium* and *Streptococcus* were predominant in the uterine microbiota and *Ureaplasma* was predominant in the vaginal microbiota of healthy primiparous cows in this study. These results are consistent with the previous study which sampled both primiparous and multiparous cows using 16S rRNA sequence analysis (35). In contrast, cows diagnosed as suffering from endometritis at 35 DPP possessed *Clostridium* dominated uterine microbiota at 0 DPP, and *Trueperella* dominated uterine microbiota on 7 and 21 DPP, and the same trend was observed in the results estimated by the culture method in this study. Pascottini et al. reported that the relative abundance of *Trueperella* in the uterine microbiota estimated by 16S rRNA sequence analysis was increased in cows with clinical endometritis (18). In addition, *Trueperella* was isolated most frequently from the uterus in cows with clinical endometritis (detection rate: 43.5%), with a particularly high number of infected cows between 9 and 15 DPP (36). These results suggest that a high prevalence of *Trueperella* in the uterus is the most important risk factor for endometritis in primiparous cows.

*Trueperella* was significantly positively correlated with *Helcococcus*, but was negatively correlated with six genera including *Lactobacillus*. These results indicate that pathogenic bacteria may interact with each other to cause inflammation, and that building an environment to encourage bacteria that are generally recognized as beneficial, such as *Lactobacillus*, may be one possible solution to reduce the abundance of *Trueperella* and *Helcococcus*. Probiotic therapies have been recognized to exhibit a beneficial effect on bacterial vaginosis in human medicine (37), indicating that administering potentially beneficial bacteria to the reproductive tract of cows may help preventing reproductive diseases.

Unique biological marker candidates within the uterine and vaginal microbiota characterizing the healthy and endometritis groups were identified in this study. In the vaginal microbiota, *Histophilus* and *Mogibacteriaceae* were unique to the pre-calving period of the endometritis group. Deng et al. reported that these two bacteria were frequently abundant in vaginal samples of non-pregnant cows by using the random forest predictive model (38). These results suggest that the abundance of these two bacteria in the vagina of pre-calving cows may be associated with the development of endometritis. In this study, *Ruminococcaceae* and *Lachnospiraceae* were more common in the vagina of healthy cows at 35 DPP. In a previous report, the vaginal microbiota of healthy cows at 7 DPP was dominated by *Ruminococcaceae*, and *Lachnospiraceae* was also a common bacterium in healthy cows (17). Other studies have shown that *Ruminococcaceae* and *Lachnospiraceae* were present in the rumen of pre-weaned calves at 2.45 and 1.60%, respectively, and in the feces at 3.04 and 2.90%, respectively, and that their occupancy increases with growth (39, 40). Since feces to vagina microbiota transfer has been shown by previous studies (16), oral administration of these bacteria (*Ruminococcaceae* and *Lachnospiraceae*) to heifers prior to fertilization may transfer to the vagina via feces and contribute to the establishment of pregnancy, calving, and subsequent stable uterine recovery.

It was found that *Enterococcus* at 0 DPP, 7 bacterial genera including *Bifidobacterium* at 28 DPP, and *Clostridium* at 35 DPP are characteristic bacteria in the uterus of the healthy group. On the other hand, *Phascolarctobacterium* and *Eubacterium* were characteristic bacteria for uterine samples of the endometritis group. *Phascolarctobacterium* induces inflammation in humans, and its high prevalence has been reported to be associated with colorectal cancer (CRC) (41). *Eubacterium* has been generally recognized as a marker of anti-inflammation in humans. However, a recent study indicated that this bacterium may also be implicated in CRC development (42).

The populations of the vaginal microbiota of cows that had given birth was clearly different from those of before birth. However, it is not clear how this altered vaginal microbiota will change following structural and functional recover of both genital tract and ovaries. Previous studies have reported the inclusion of dystocia as a risk factor for metritis and endometritis (43, 44). In the present study, the presence of endometritis tended to be associated with the dystocia score (Mann-Whitney *U*-test, *p* = 0.057), suggesting that dystocia was indirectly associated with bacterial composition observed in endometritis group in this study.

In the current study, we analyzed microbiota of both uterus and vagina in the early postpartum period and identified unique bacterial genera that are characteristic of healthy and endometritis groups. Since the abundance and correlation between pathogenic bacteria, including *Trueperella*, exhibited similar trends to previous reports which did not discriminate primiparous and multiparous cows, the effect of parity on pathogenic bacterial colonization dynamics is thought to be small. However, in this study, differentiating primiparous cows has allowed us to characterize the bacterial dynamics in healthy cows which has not been previously reported. These findings could be useful for predicting endometritis and for developing prevention or treatment strategies. However, there are several limitations in our study: all primiparous cows came from a single farm, the sample size was small, and clinical and sub-clinical endometritis could not be analyzed separately. Therefore, our findings may be difficult to extrapolate to different regions or different cattle types. All primiparous cows used in this study was received AI or ET to be pregnant (no natural breeding), therefore the effect of the penile microbiota of the bull, which may be transferred by natural breeding, was not taken into account. The presence of microbiota in semen has become clearer in recent years (45). In this study, no single type semen was used in AI, but the microbiota in each semen was not analyzed. The complex interactions (e.g., natural breeding, AI or ET, the microbiota of the semen used in AI, the degree of recovery of the uterus and ovaries after parturition) should be considered when assessing reproductive tract microbiota.

## Data Availability Statement

The original contributions presented in the study are publicly available. This data can be found here: https://www.ddbj.nig.ac.jp/ BioProject: PRJDB11963.

## Ethics Statement

The animal study was reviewed and approved by Rakuno Gakuen University under protocol number VH16C7.

## Author Contributions

HK, TS, and MU: conceptualization. HK: methodology, investigation all experiments, and writing original draft. HK and TS: sample collection. HK: bacterial culture. HK and SH: 16S rRNA analysis. KO, MT, and MU: funding acquisition and resources. SK and YT: supervision. All authors contributed to the article and approved the submitted version.

## Conflict of Interest

HK, SH, KO, MT, and SK are employed by Miyarisan Pharmaceutical Co., Ltd. The remaining authors declare that the research was conducted in the absence of any commercial or financial relationships that could be construed as a potential conflict of interest.

## Publisher's Note

All claims expressed in this article are solely those of the authors and do not necessarily represent those of their affiliated organizations, or those of the publisher, the editors and the reviewers. Any product that may be evaluated in this article, or claim that may be made by its manufacturer, is not guaranteed or endorsed by the publisher.
