# Supplementary material for: Characterization of Reproductive Microbiota of Primiparous Cows During Early Postpartum Periods in the Presence and Absence of Endometritis
Source: Front Vet Sci. 2021 Oct 18;8:736996. doi: 10.3389/fvets.2021.736996 (PMC8558311; doi:10.3389/fvets.2021.736996)
Supplement: Supplementary file 1 [file Data_Sheet_1.PDF]

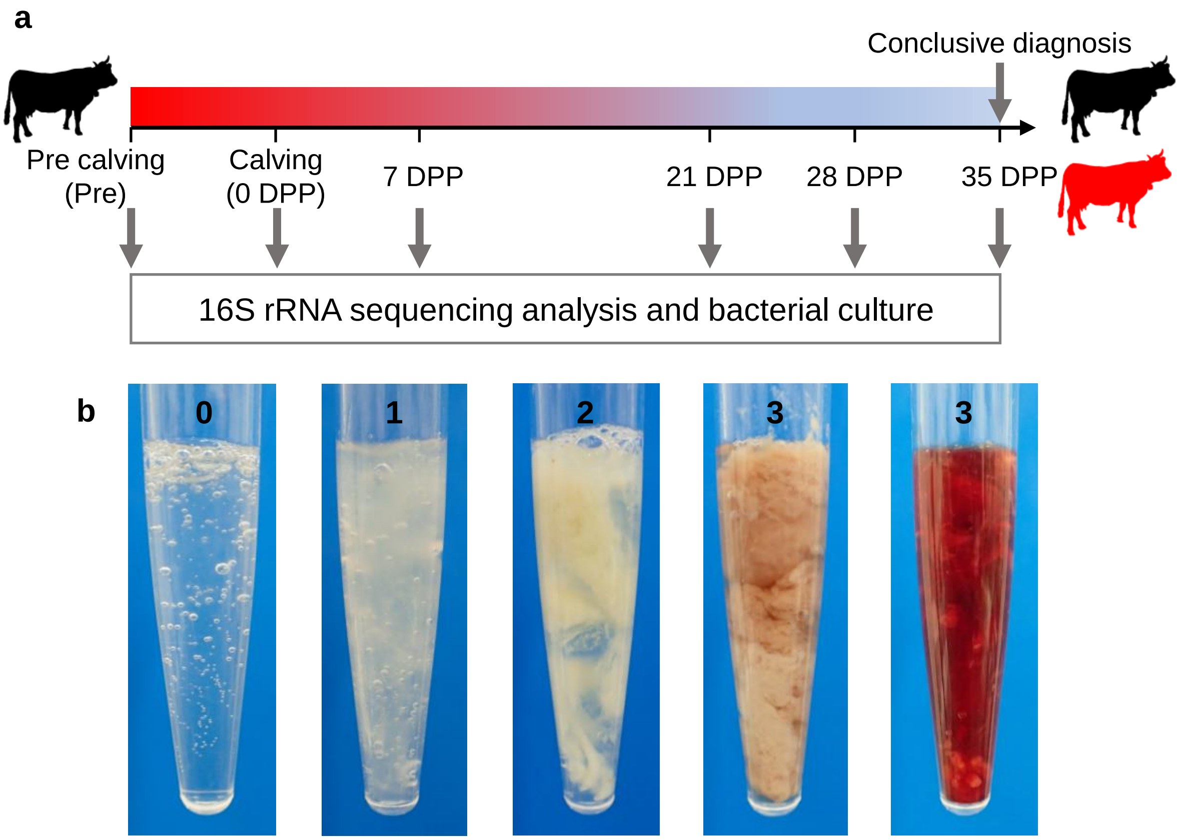

c

Evaluation items	Classifications		Score
Vaginal discharge	Discharge containing ≥ 50 % purulent material		3
	Discharge containing < 50 % purulent material		2
	Mucus containing flecks of white or off-white pus		1
	Clear or translucent mucus		0
The diameter of largest uterine horn	Large	≥ 5.5 cm	2
	Medium	More than 3.5 to less than 5.5 cm	1
	Small	≤ 3.5 cm	0
The diameter of largest uterine cervix	Large	≥ 7.0 cm	2
	Medium	More than 4.5 to less than 7.0 cm	1
	Small	≤ 4.5 cm	0

The total score was discribed as following; 6–7 = Severe, 4–5 = Moderate, 1–3 = Mild, 0 = Normal

Supplementary Figure 1 | Sampling periods and diagnostic criteria for clinical endometritis in postpartum primiparous cows.
(a) Schematic illustration of the experimental design. Each cow was diagnosed by a series of gynecological examinations after 21 DPP, and conclusive diagnosis was done at 35 DPP. Samples were collected at indicated time points. (b) Vaginal mucus character was graded as 0 = clear or translucent mucus; 1 = mucus containing flecks of white or off-white pus; 2 = exudate containing < 50% white or off-white mucopurulent material; and 3 = exudate containing ≥ 50% purulent material, usually white or yellow, but occasionally sanguineous. (c) Conclusive diagnostic criteria on 35 DPP. The total score was described as the following; 6–7 = Severe, 4–5 = Moderate, 1–3 = Mild, 0 = Normal.

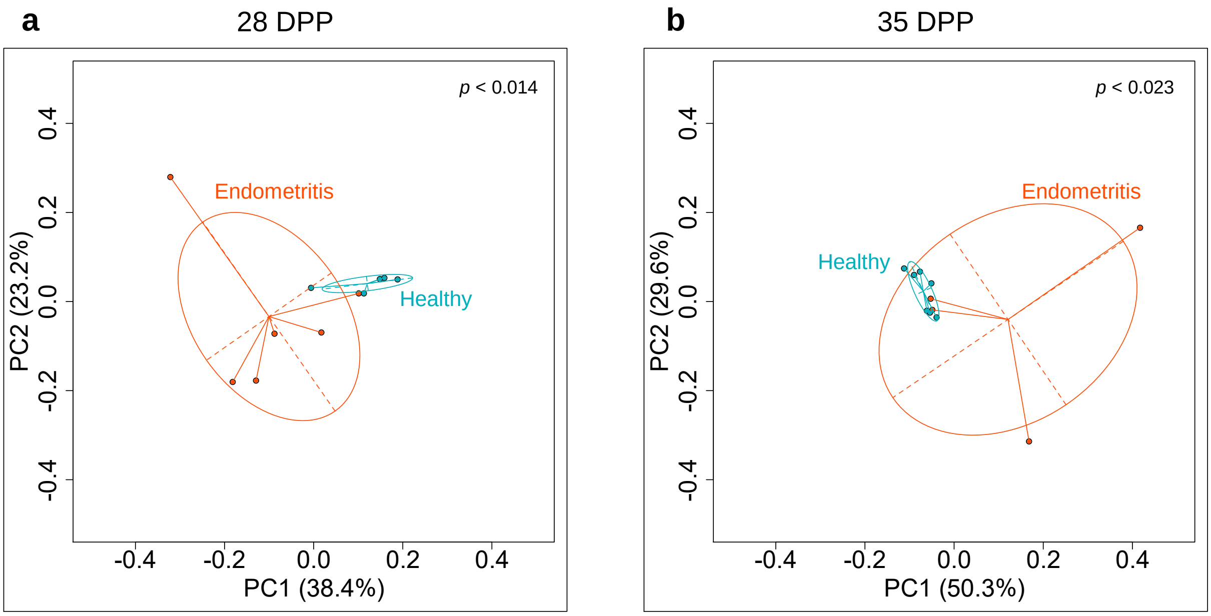

Supplementary Figure 2 | Beta-diversity of uterine microbiota as shown by 16S rRNA gene sequence.
(a-b) Principal Coordinate Analysis (PCoA) based on weighted UniFrac distance of uterine microbiota at 28 DPP (a) and 35 DPP (b). Statistical calculation was done using pairwise PERMANOVA with 999 permutations.

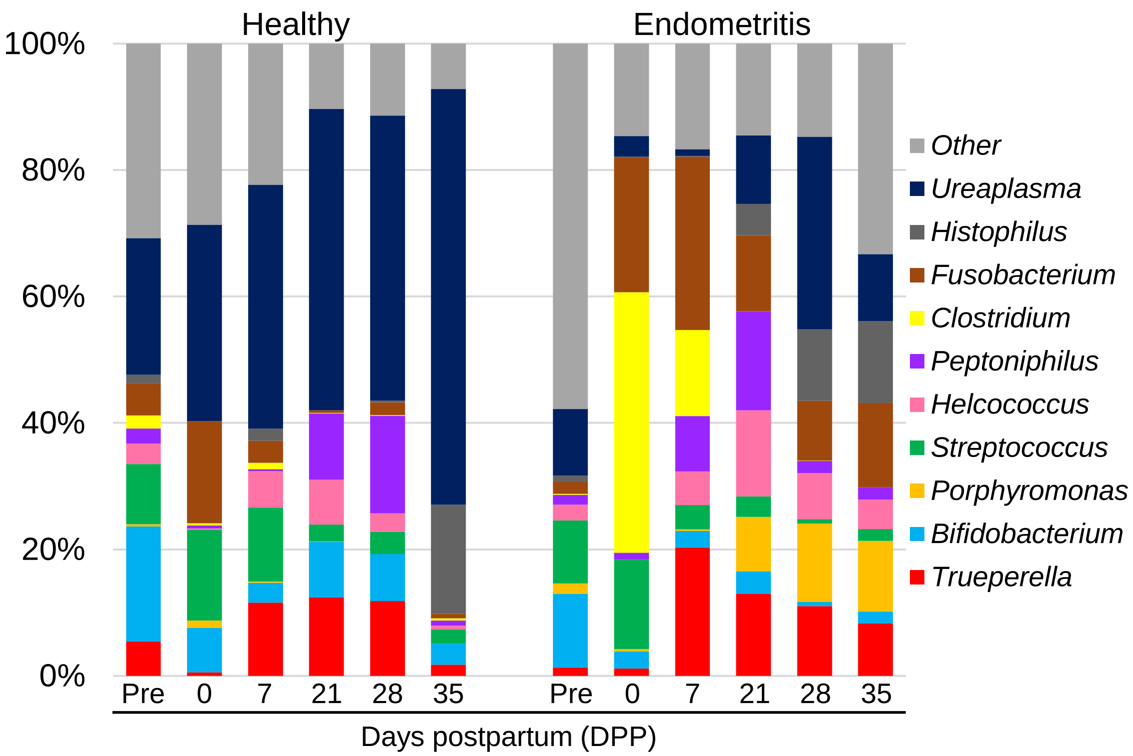

Supplementary Figure 3 | Stacked bar chart showing the mean relative abundant of top 10 bacterial genera in vaginal microbiota.
Profiles demonstrated dynamic change of vaginal microbiota with time and depicted separation between healthy and endometritis groups.

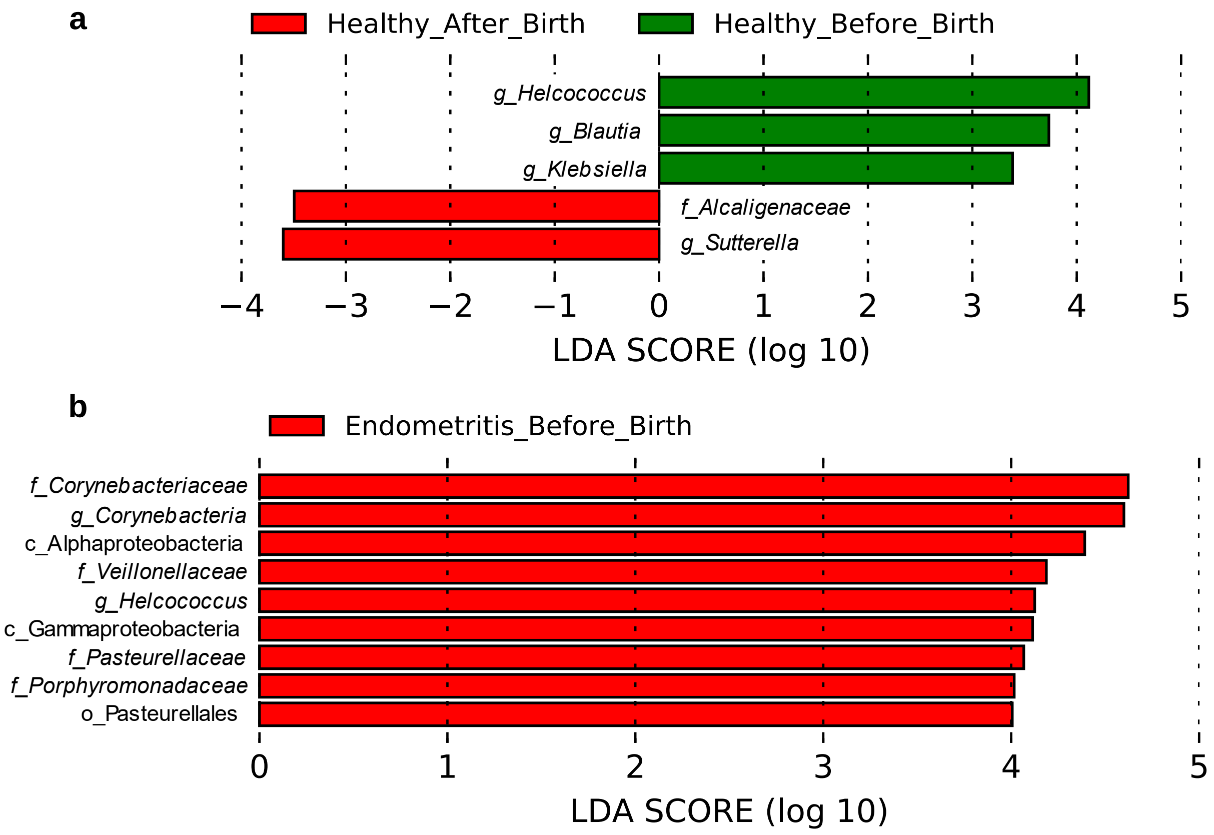

Supplementary Figure 4 | Effect of calving on bacterial genera in vaginal microbiota.

Different structures of vaginal microbiota in the ‘before birth’ group (Pre) and ‘after birth’ group (0 DPP) for both healthy (a) and endometritis cows (b). g_ means genus level. f_, o_, and c_ indicate classification only at the family, order, and class level with genus not precisely defined.
